# Daily life physical activity in patients with chronic stage IV sarcoidosis: A multicenter cohort study

**DOI:** 10.1002/hsr2.109

**Published:** 2019-01-15

**Authors:** Sarah Froidure, Maeva Kyheng, Jean Marie Grosbois, Francois Lhuissier, Sandrine Stelianides, Lidwine Wemeau, Benoit Wallaert

**Affiliations:** ^1^ Service de Pneumologie et ImmunoAllergologie, Centre de compétence des maladies rares and Univ. Lille CHU Lille Lille France; ^2^ Department of Biostatistics CHU Lille Lille France; ^3^ FormAction Santé, Home‐based pulmonary rehabilitation team, rue Pietralunga Pérenchies France; ^4^ Sorbonne Paris Cité, Laboratoire Hypoxie et Poumon Université Paris 13 Bobigny France; ^5^ Assistance Publique – Hôpitaux de Paris, Hôpital Avicenne Service de Physiologie, explorations fonctionnelles et médecine du sport Bobigny France; ^6^ Division of Pneumology, Bichat Hospital, APHP Paris‐Diderot University Paris France

**Keywords:** aerobic capacity, anxiety, daily life physical activity, depression, fatigue, pulmonary function test, sarcoidosis

## Abstract

**Background and objectives:**

Little is known about the consequences of chronic sarcoidosis on daily life physical activity (DL_PA_). The aim of this prospective study was to measure DL_PA_ in patients with chronic sarcoidosis and to determine its relationship to clinical and functional parameters.

**Methods:**

Fifty‐three patients with chronic sarcoidosis and 28 healthy control subjects were enrolled in this multicenter prospective study. Two markers of DL_PA_ (number of steps walked per day [SPD]) and total daily energy expenditure (TEE) were assessed for five consecutive days with a physical activity monitor. Pulmonary function, aerobic capacity (maximal oxygen uptake [VO_2_max]), exercise capacity (6‐min walk test [6MWT]), and quality of life (self‐reported questionnaires) were also evaluated. Comparisons of DL_PA_ parameters between the two groups were performed using an analysis of covariance adjusted for age, sex, and body mass index (BMI). Relationships between DL_PA_ parameters and patient characteristics were assessed in multivariable linear regression models.

**Results:**

Patients with sarcoidosis walked significantly fewer SPD than did the control subjects (6395 ± 4119 and 11 817 ± 3600, respectively; *P* < 0.001 after adjustment for age, BMI, and sex). TEE was not significantly different between patients with sarcoidosis and healthy controls (median [interquartile range]: 2369 [2004‐2827] and 2387 [2319‐2876] kcal/day, respectively, *P* = 0.054 adjusted for age, BMI, and sex). SPD showed significant positive correlations with 6MWT distance (Pearson's correlation, *r* = 0.32, 95% confidence intervals [95%CI] = 0.06, 0.55; *P* = 0.019), VO_2_max (*r* = 0.44, 95%CI = 0.17, 0.65; *P* = 0.002), and Visual Simplified Respiratory Questionnaire score (*r* = 0.44, 95%CI = 0.19, 0.64; *P* = 0.001), and a significant negative correlation with modified Medical Research Council questionnaire score (*r* = −0.38, 95%CI = −0.60, −0.10; *P* = 0.009). TEE was significantly correlated with BMI (*r* = 0.38, 95%CI = 0.13, 0.59; *P* = 0.004), forced expiratory volume in 1 second (*r* = 0.55, 95%CI = 0.33, 0.71; *P* < 0.001), total lung capacity (*r* = 0.44, 95%CI = 0.18, 0.64; *P* = 0.001), and forced vital capacity (*r* = 0.56, 95%CI = 0.34, 0.72; *P* < 0.001). In multivariable analysis, SPD remained associated only with VO_2_max.

**Conclusion:**

Patients with chronic sarcoidosis appear to have reduced DL_PA_ mainly because of compromised VO_2_max.

## 1 INTRODUCTION

Sarcoidosis is a systemic disease of unknown cause that can affect many organs but that most frequently (90%‐95% of cases) affects the lungs.^1^ Together, sarcoidosis and idiopathic pulmonary fibrosis represent the two most common etiologies of interstitial lung disease.^1^

Histologically, sarcoidosis is characterized by the formation of epithelioid and giant cell granulomas without caseous necrosis,^2^ and it is usually classified in five stages based on radiological findings. Stage IV corresponds to the chronic fibrosing form of the disease, which accounts for approximately 5.4% of pulmonary sarcoidosis cases.^3^ Patients with chronic respiratory diseases often display disabling dyspnea associated with a progressive reduction in daily life physical activity (DL_PA_), as has previously been demonstrated in patients with interstitial lung diseases, including idiopathic pulmonary fibrosis.^4^ Reduced physical activity is an important clinical parameter related to increased morbidity, mortality, and hospitalizations in many chronic diseases.^5^

Little is known about DL_PA_ in patients with chronic sarcoidosis.^6^, ^7^, ^8^, ^9^ Saligan showed a reduction in physical activity associated with fatigue, depressive symptoms, and a shorter distance in the 6‐minute walk test (6MWT),^9^ whereas Bahmer et al found a significant association with 6MWT distance and quality of life scores but only a weak association with fatigue.^6^ However, these studies examined patients with a variety of respiratory conditions, and relatively few of them had chronic fibrotic disease. With this in mind, we sought to quantify DL_PA_ in patients with stage IV chronic sarcoidosis and determine the relationships between two‐defined DL_PA_ parameters and a number of pulmonary function, aerobic capacity, and quality of life measures. The main objective of this work was to evaluate DL_PA_ in patients with chronic stage IV sarcoidosis compared with healthy control subjects. The secondary objective was to determine the factors associated with DL_PA_ in patients with chronic sarcoidosis.

## 2 METHODS

### 2.1 Patients

Fifty‐three patients with stage IV chronic sarcoidosis^10^ were enrolled in the study. Of these, 29 were part of the National PHRC 2012‐A00347‐36 “Respiratory rehabilitation in chronic fibrotic sarcoidosis (stage IV): a randomized therapeutic trial” (ClinicalTrials.gov NCT02044939), and 24 consecutive patients were referred by their pulmonologists in the North of France to our Center for Rare Pulmonary Diseases for prerehabilitation assessment. Inclusion criteria were: (1) a sarcoidosis diagnosis according to the American Thoracic Society (ATS)/European Respiratory Society (ERS)/World Association of Sarcoidosis and Other Granulomatous Disorders (WASOG) statement^10^ and (2) radiographic stage IV disease defined by patent advanced fibrosis with evidence of upper lobe volume loss with hilar retraction with or without masses, coarse linear bands, honeycombing, bullae, and emphysema. In addition, we enrolled 28 healthy volunteers who were students or relatives of employees at the hospital. The controls were selected to be comparable in age and sex ratio. All control subjects had normal spirometry results. None of the patients or control subjects was engaged in exercise training programs prior to the study. All individuals gave informed consent, and approval for the use of the data was provided by the Institutional Review Board of the French Learned Society for Pulmonology (CEPRO 2017‐007).

### 2.2 Assessment of daily life physical activity

Subjects were equipped with a physical activity monitor (SenseWear Pro armband and SenseWear software version 8.0; BodyMedia Inc., Pittsburgh, Pennsylvania, United States) and instructed to wear the device continuously, except while showering or bathing, for five consecutive days (three weekdays and two weekend days). The device was positioned on the upper right arm at the midpoint between the acromion and the olecranon, as previously described.^11^ DL_PA_ was assessed by measuring the number of steps per day (SPD) and the total daily energy expenditure (TEE, in kcal/day). All functional tests and questionnaires were performed on the same day, prior to the 5‐day DL_PA_ monitoring.

### 2.3 Pulmonary function tests

Forced vital capacity (FVC), forced expiratory volume in 1 second (FEV_1_), and total lung capacity (TLC) were measured by spirometry and plethysmography with a Jaeger‐Masterlab cabin (Vyaire Medical, Hoechberg, Germany), and single‐breath diffusing capacity of the lung for carbon monoxide (DLco, in mL CO/min/mmHg) was measured and corrected for hemoglobin concentration. Reference equations for lung volumes and DLco were taken from ERS.^12^, ^13^ The lower limits of “normal” were set at the fifth percentile (or predicted minus 1.64 standard deviations [SD]) of each reference population, according to the 2005 ATS/ERS guidelines.^14^ The results are conventionally expressed as percent of the predicted values.

### 2.4 Six‐minute walk test (6MWT)

The 6MWT was performed in accordance with international recommendations^15^ using a 30‐m indoor corridor in our hospital. Two 6MWT were performed and the results of the second ISWT were recorded for analysis. Pulse O_2_ saturation (SpO_2_) and heart rate were monitored continuously using a Novametrix 513 Pulse Oximeter (Wallingford, Connecticut, United States).

### 2.5 Cardiopulmonary exercise test

Subjects completed a triangular exercise test on a cycle ergometer (Ergometrics 800; Ergoline, Bitz, Germany), with blood pressure and electrocardiographic monitoring (Medcard; Medisoft, Sorrine, Belgium) according to a standardized protocol, as detailed previously.^16^ We focused on aerobic capacity assessed by maximal oxygen uptake (VO_2_max), and the results are expressed as mL O_2_/kg/min and the percentage of predicted values.^17^

### 2.6 Dyspnea

Dyspnea occurring during the patients' daily lives was assessed using the modified Medical Research Council (mMRC) self‐administered questionnaire, which consists of five questions about perceived breathlessness and is scored on a scale from 0 (not troubled by breathlessness except during strenuous exercise) to 4 (very severe dyspnea: too breathless to leave the house or breathless when dressing or undressing).^18^

### 2.7 Fatigue

Fatigue occurring during the patients' daily lives was assessed using the Fatigue Assessment Scale (FAS) questionnaire, in which a score greater than 22 (on a scale of 10‐50) is considered clinically significant.^19^

### 2.8 Quality of life

The patients indicated their overall quality of life using the Visual Simplified Respiratory Questionnaire (VSRQ). A score of at least 80 (on a scale of 0‐100) indicates a satisfactory quality of life.^20^

### 2.9 Anxiety and depression

The Hospital Anxiety and Depression Scale (HADS) was designed to identify and quantify the two most common forms of psychological disorders in medical patients.^21^ For both subscales, a score of 8 to 10 (on a scale of 0‐21) is indicative of uncertain symptoms, and a score greater than 11 is indicative of clinically relevant symptoms.

### 2.10 Statistical analysis

Continuous variables are expressed as means (SD) for normally distributed data and medians (interquartile range [IQR]) for other data. Categorical variables are expressed as numbers (percentage). Normality of distribution was assessed visually using histograms and statistically using the Shapiro–Wilk test. Patient characteristics were compared with those of healthy control subjects using student's *t* test for quantitative variables and Chi‐square test for sex. DL_PA_ parameters were compared between patients and control subjects using an analysis of variance. Comparisons were further adjusted for prespecified confounding factors (age, body mass index [BMI], and sex) using multivariable linear regression models. For the patient group, correlations between each DL_PA_ parameter were assessed by calculating Pearson's correlation coefficients (r) with 95% confidence intervals (95%CIs) calculated using Fisher's Z transformation. Associations between DL_PA_ parameters and patient characteristics (continuous variables) were first assessed in univariable analyses by calculating Pearson's correlation coefficients. Significant characteristics (*P* < 0.1) were then entered into a forward‐stepwise multivariable linear regression analysis by including prespecified confounding factors (age, BMI, and sex) as forced variables. Colinearity between candidate factors in the multivariable analysis was examined by calculating variance inflation factors using an alert threshold value of 2.5.^22^ Finally, the selected multivariable model was further adjusted for the prespecified confounding factors (age, BMI, and sex). Statistical testing was done at the two‐tailed *α* level of 0.05. Data were analyzed using SAS software version 9.4 (SAS Institute, Cary, North Carolina, United States).

## 3 RESULTS

The characteristics of the patients with sarcoidosis and healthy control subjects are summarized in Tables 1 and Table 2. Pulmonary function tests showed that DLco was reduced (<80% predicted) in 46 of the 53 (87%) patients with sarcoidosis. A restrictive pattern (TLC < 80% predicted) was observed in 20 patients (38%), and an obstructive pattern (FEV_1_/FVC < 70%) was seen in 31 patients (58%). Twenty‐two patients (41%) displayed significant fatigue (FAS score > 22), 14 (26%) had a significant HADS anxiety score (≥11), and 11 (20%) had a significant HADS depression score (≥11). Most of the patients (75.5%) were treated with steroids (mean daily dose 11.3 mg/day); of these, 45.3% were also taking additional immunosuppressive drugs.

**Table 1:** Characteristics of patients with sarcoidosis and healthy controls

Characteristic	Patients with Sarcoidosis	Healthy Controls	*P* Value
	N = 53	N = 28	
Age (years) (mean [SD])	59.2 (9.4)	57.1 (9.4)	0.35^a^
BMI (kg/m^2^) (mean [SD])	28.1 (6.6)	23.8 (2.7)	< 0.001^a^
Women (n [%])	24.0 (47.3)	16.0 (57.1)	0.31^b^
Patients taking prednisone (n [%])	40.0 (75.5)	‐	‐
Prednisone dose (mg/day) (mean [SD])	11.3 (11.2)	‐	‐
Patients taking additional immunosuppressant drugs (n [%])	24.0 (45.3)	‐	‐

a Calculated using student's *t* test.

b Calculated using Chi‐square test. BMI: body mass index; SD: standard deviation.

**Table 2:** Functional characteristics of the 53 patients with sarcoidosis

	Values	
Characteristic	Mean (SD)	Median (IQR)
FEV_1_ (L)	1.8 (0.7)	1.7 (1.3, 2.2)
FEV_1_ (% predicted)	63.5 (19.6)	61.0 (51.0, 72.0)
TLC (L)	5.0 (1.5)	4.9 (4.0, 5.8)
TLC (% predicted)	83.2 (18.6)	84.0 (71.0, 93.0)
FVC (L)	2.8 (1.0)	2.5 (2.1, 3.4)
FVC (% predicted)	79.4 (19.5)	75.0 (66.0, 96.0)
FEV_1_/FVC (%)	80.6 (16.4)	80.6 (69.6, 96.8)
DL_CO_ (mL/min/mmHg)	12.3 (6.2)	11.2 (7.1, 16.7)
DL_CO_ (% predicted)	55.7 (18.4)	57.0 (42.0, 65.0)
6MWT distance (m)	423 (115)	441 (360, 490)
6MWT nadir SpO_2_ (%)	90.2 (6.1)	92.0 (89.0, 95.0)
VO_2_max (mL/kg/min)	17.6 (5.7)	15.5 (13.9, 21.5)
VO_2_max (% predicted)	71.3 (18.1)	69.0 (61.5, 80.5)
VSRQ score	41.7 (16.4)	40.5 (31.0, 54.5)
HADS anxiety score	8.2 (4.0)	7.5 (5.0, 10.5)
HADS depression score	6.8 (4.0)	6.5 (4.0, 9.5)
mMRC score	2.0 (1.0)	2.0 (1.0, 3.0)
FAS score	26.2 (6.6)	26.0 (22.0, 29.0)

Abbreviations: 6MWT: 6‐minute walk test; DLco: diffusing capacity for carbon monoxide; FAS: Fatigue Assessment Score; FEV_1_: forced expiratory volume in 1 second; FVC: forced vital capacity; VSRQ: Visual Simplified Respiratory Questionnaire; HADS: Hospital Anxiety and Depression Scale; mMRC: modified Medical Research Council questionnaire; SD: standard deviation; TLC: total lung capacity; IQR: interquartile range.

**Figure 1:**
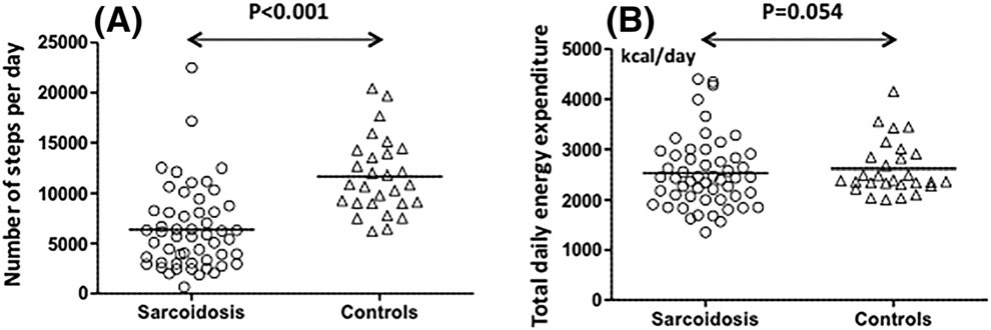
Physical activity in daily life for patients with sarcoidosis and healthy controls (A) Number of steps per day and (B) total daily energy expenditure in healthy controls (n = 28) and patients with chronic sarcoidosis (n = 53). Lines indicate mean. Differences were analyzed using student's *t* test.

The DL_PA_ parameters for patients with sarcoidosis and control subjects are shown in Table 3 and Figure 1. Patients walked significantly fewer SPD than did the control subjects (6395 ± 4119 and 11 817 ± 3600, respectively; *P* < 0.001 after adjustment for age, BMI, and sex in a multivariable linear regression model). No statistically significant difference was observed for TEE between patients with sarcoidosis and healthy controls (median [IQR] 2369 [2004‐2827] vs 2387 [2319‐2876] kcal/day, respectively; *P* = 0.054 after adjustment for age, BMI, and sex in a multivariable linear regression model). There was a moderate correlation between the DL_PA_ parameters (Pearson's correlation [r] = 0.49, 95%CI = 0.25, 0.67; *P* < 0.001). In addition, the time (min/day) spent in activities with an estimated energy expenditure of greater than 2.5 metabolic equivalents (METs) and the total energy expenditure greater than 2.5 METs (kcal/day) were significantly lower in patients than in controls (Supplementary table 1).

**Table 3:** Daily life physical activity (DL_PA_) parameters in patients with sarcoidosis and healthy control subjects

Parameter	Patients with Sarcoidosis N = 53	Healthy Controls N = 28	*P* Value^a^	Adjusted *P* Value^b^
Number of steps per day (mean [SD])	6395 (4119)	11 817 (3600)	<0.001	<0.001
Total energy expenditure, kcal/day (median [IQR])	2369 (2004, 2827)	2387 (2319, 2876)	0.14^c^	0.054^c^
				

a Calculated using student's *t* test.

b Calculated using multivariable linear regression analysis adjusted for age, sex, and body mass index (BMI).

c After log transformation. IQR: interquartile range; SD: standard deviation.

Table 4 shows the correlations between DL_PA_ parameters and pulmonary function tests, exercise tests, self‐reported questionnaires, and the main characteristics of the patients with sarcoidosis. Oral steroids did not influence the DL_PA_ parameters (data not shown). In univariable analysis, SPD was positively correlated with 6MWT distance (*r* = 0.32, 95%CI = 0.06, 0.55; *P* = 0.019), VO_2_max (*r* = 0.44, 95%CI = 0.17, 0.65; *P* = 0.002), and VSRQ score (*r* = 0.44, 95%CI = 0.19, 0.64; *P* = 0.001), and negatively correlated with mMRC score (*r* = −0.38, 95%CI = −0.60, −0.10; *P* = 0.009). In multivariable analysis, only VO_2_max remained significantly associated with SPD (*P* = 0.012 after adjustment for age, BMI, and sex in a multivariable linear regression model; Table 5). TEE was positively correlated with BMI (*r* = 0.38, 95%CI = 0.13, 0.59; *P* = 0.004), FEV_1_ (*r* = 0.55, 95%CI = 0.33, 0.71; *P* < 0.001), TLC (*r* = 0.44, 95%CI = 0.18, 0.64; *P* = 0.001), and FVC (*r* = 0.56, 95%CI = 0.34, 0.72; *P* < 0.001) in univariable analysis. Since the difference in TEE was considered not to be clinically significant, the multivariable analysis were not performed.

**Table 4:** Correlations between daily life physical activity (DL_PA_) parameters and characteristics in the patients with sarcoidosis (n = 53)

Characteristic	Number of Steps Per Day		Total Energy Expenditure (kcal/day)^a^	
	*r* b (95%CI)	*P* value	*r* b (95%CI)	*P* value
Age	−0.21 (−0.45, 0.06)	0.13	−0.06 (−0.32, 0.22)	0.69
BMI	−0.10 (−0.36, 0.17)	0.47	0.38 (0.13, 0.59)	0.004
Prednisone dose^c^	−0.22 (−0.46, 0.05)	0.11	−0.13 (−0.39, 0.14)	0.34
FEV_1_	0.23 (−0.04, 0.47)	0.096	0.55 (0.33, 0.71)	<0.001
FVC	0.26 (−0.01, 0.50)	0.056	0.56 (0.34, 0.72)	<0.001
FEV_1_/FVC	−0.04 (−0.31, 0.23)	0.77	0.05 (−0.22, 0.32)	0.71
TLC	0.21 (−0.07, 0.46)	0.14	0.44 (0.18, 0.64)	0.001
DLco	−0.05 (−0.33, 0.22)	0.69	0.18 (−0.10, 0.44)	0.21
VO_2_max	0.44 (0.17, 0.65)	0.002	0.04 (−0.25, 0.33)	0.77
6MWT distance	0.32 (0.06, 0.55)	0.019	0.14 (−0.14, 0.39)	0.33
6MWT nadir SpO_2_	0.27 (−0.004, 0.50)	0.053	0.17 (−0.11, 0.43)	0.22
mMRC score	−0.38 (−0.60, −0.10)	0.009	−0.13 (−0.40, 0.16)	0.37
HADS score, depression	−0.19 (−0.44, 0.08)	0.17	−0.01 (−0.28, 0.26)	0.95
HADS score, anxiety	−0.004 (−0.28, 0.27)	0.97	−0.007 (−0.27, 0.28)	0.96
FAS score	0.11 (−0.21, 0.40)	0.49	0.09 (−0.23, 0.38)	0.59
VSRQ score	0.44 (0.19, 0.64)	0.001	−0.007 (−0.28, 0.27)	0.96

a After log transformation.

b Pearson's correlation coefficient.

c Dose in mg/day.

Abbreviations: 6MWT: 6‐minute walk test; BMI: body mass index; CI: confidence interval; DLco: diffusing capacity for carbon monoxide; FAS: Fatigue Assessment Score; FEV_1_: forced expiratory volume in 1 second; FVC: forced vital capacity; HADS: Hospital Anxiety and Depression Scale; mMRC: modified Medical Research Council questionnaire; TLC: total lung capacity; VSRQ: Visual Simplified Respiratory Questionnaire.

**Table 5:** Final model of factors affecting the number of steps per day in patients with sarcoidosis after adjustment for age, sex, and body mass index (BMI)

Parameters and Factors	Estimate^a^	SE^a^	Partial *R* ^*2*^ (%)^a^	*P* Value^a^
Number of steps per day			13.3	
VO_2_max	314	120		0.012
Age	−47.7	61.8		0.44
Sex (men vs women)	−221	1285		0.86
BMI	28.2	101		0.78

a All variables associated with number of steps per day in the univariable analyses (at *P* < 0.10) were considered as candidate variables for multivariable analysis. The multivariable analysis was conducted using a forward‐stepwise selection approach, as specified in the statistical analysis section, by including pre‐specified confounders as forced variables (age, BMI, and sex).

## 4 DISCUSSION

The results of this study show that DL_PA_ is decreased in adult patients with stage IV sarcoidosis and that SPD is significantly associated with VO_2_max. Our results are in agreement with three previous studies evaluating physical activity in patients with sarcoidosis.^6^, ^7^, ^8^ Kostorz et al^7^ evaluated the comorbidities associated with DL_PA_ in 30 patients with sarcoidosis, albeit without precise radiological staging and found that the patients had walked a mean (±SD) SPD of 5214 (±2699). Pilzak et al found that patients with sarcoidosis not only showed low mean SPD (4566 ± 2378) but also displayed reduced exercise tolerance, as measured by the VO_2_max and 6MWT.^8^ However, that study did not examine correlations between DL_PA_ and pulmonary function, exercise tolerance, or mood parameters (anxiety and depression). In our study, multivariable analysis showed no correlation between SPD and resting functional parameters (DLco, FEV_1_, TLC, and FVC), and a correlation between SPD and exercise tolerance was detected only when the latter was evaluated by VO_2_max but not by the 6MWT. Finally, the study by Bahmer et al showed an association between SPD and 6MWT distance, fatigue score (Multidimensional Fatigue Inventory), and quality of life scores (St. George's Respiratory and SF‐12 questionnaires).^6^ However, it is important to note that Bahmer et al included only three patients with chronic sarcoidosis stage IV in their study cohort, which could explain the differences between their and our results. Indeed, the mean (±SD) SPD recorded in that study (7490 ± 3007)^6^ was higher than in our study (6395 ± 4119).

Our data suggest that submaximal stress tests are insufficient to evaluate the mechanisms underlying the reduced DL_PA_ in patients with sarcoidosis. To our knowledge, only one study has previously evaluated parameters other than SPD to quantify DL_PA_ in patients with chronic sarcoidosis.^7^ Although daily energy expenditure was also associated with VO_2_max in that study, only patients with less severe disease (stage II) were evaluated.^7^ We also found that total daily expenditure was correlated with FEV_1_. Bahmer et al^6^ found that FEV_1_ was associated with the 6MWT distance but not with SPD in patients with sarcoidosis. However, the mean FEV_1_ in that study was less severe than in our study. Similar findings to ours have been reported in patients with chronic obstructive pulmonary disease, who display a progressive decrease in FEV_1_ that is paralleled by a decrease in physical activity.^23^ Our study is the first to show that the obstructive ventilatory defect is correlated with DL_PA_ in patients with sarcoidosis and might explain part of the reduction in daily TEE. Interestingly, both the pulmonary function pattern and the high percentage of patients with an obstructive ventilatory defect in our cohort are similar to those reported by Nardi et al, who examined a large group of patients with stage IV sarcoidosis.^24^

Although patients with sarcoidosis commonly show fatigue^25^ associated with both a poor quality of life^26^ and a shorter 6MWT distance,^6^, ^27^ we did not find a relationship between fatigue and DL_PA_ in our study_._ We also did not observe an association between DL_PA_ and either anxiety or depression scores, which, to our knowledge, is an observation not previously reported for patients with sarcoidosis. Our finding of only weak/moderate associations between DL_PA_ parameters and quality of life contrasts with the studies of Bahmer et al^6^ and Baughman et al,^28^ who found strong correlations between the St George's Respiratory Questionnaire (SGRQ) score and the SPD and 6MWT distance, respectively. The altered quality of life in patients with sarcoidosis could well be a consequence and not a causal determinant of the limitation of DL_PA._ Indeed, one can hypothesize that functional impairment (ie, FEV_1_ and VO_2_max) is more important than fatigue in explaining limitations of DL_PA_ and low quality of life in patients with sarcoidosis. Nevertheless, it is important to note that VSRQ and SGRQ are not routinely used to assess patients with sarcoidosis, so these tools may be insufficiently specific to evaluate quality of life in our population.

Only a fraction of the reduction in DL_PA_ in patients with sarcoidosis could be explained by the resting and exercise functional parameters examined here, suggesting that it may be important to investigate the influence of environmental and sociodemographic factors in future studies. In support of this, a study of patients with chronic obstructive pulmonary disease found that dog ownership or having small children was significantly associated with time spent in moderate to sustained physical activities (3‐6 metabolic equivalents).^29^ Place of residence and psychological factors also seem to be important influences on the level of physical activity. Sallis et al found an association between daily physical activity in healthy adults, as measured by an accelerometer and the density of neighborhood parks and public transport.^30^ However, there have been no comparable studies in patients with respiratory diseases. Bauman et al reviewed 16 articles on healthy adults with a history of physical activity during adolescence and adulthood, and they found that self‐efficacy (confidence in the ability to be active in specific situations) is the most important determinant of physical activity.^31^ These different sociodemographic elements must be studied in various patient populations to determine their relevance to each specific respiratory disease and their contributions to interindividual variability in DL_PA_.

In addition to the limitations inherent to the observational exploratory design, our study has several weaknesses. There was no formal sample size calculation, so we cannot exclude the possibility that differences may have been overlooked because of inadequate statistical power. In a posterior power calculation, the smallest significant difference that our sample size (53 patients with sarcoidosis and 28 healthy controls) allowed us to detect with 80% power was 0.7 (standardized mean difference), which is interpreted as a large effect size.^32^ The lack of a validation dataset was also a limitation. Additionally, we also cannot exclude response bias in the questionnaires.

## 5 CONCLUSION

DL_PA_ is reduced in patients with chronic stage IV sarcoidosis compared with control subjects. Our findings implicate VO_2_max, which explains 13.3% of SPD, as significant influence on DL_PA_ in these patients. However, pulmonary function tests were insufficient to evaluate DL_PA_ in our study, and other parameters must be evaluated to understand all determinants of DL_PA_ in patients with chronic sarcoidosis. Future studies should also evaluate how DL_PA_ changes during and after pulmonary rehabilitation.

## CONFLICTS OF INTEREST

For each author, no significant conflicts of interest exist with any companies or organizations whose products or services are mentioned in this article.

## AUTHOR CONTRIBUTIONS

Conceptualization: Benoit Wallaert, Jean Marie Grosbois

Formal analysis: Maeva Kyheng, Sarah Froidure

Investigation: Benoit Wallaert, Jean Marie Grosbois, Francois Lhuissier, Sandrine Stelianides, Lidwine Wemeau

Writing—original draft: Sarah Froidure

Writing—review and editing: Benoit Wallaert

## Supporting information

Table S1. Time spent in activities requiring at least 2.5 METs
