# Supplementary material for: Daily life physical activity in patients with chronic stage IV sarcoidosis: A multicenter cohort study
Source: Health Sci Rep. 2019 Jan 15;2(2):e109. doi: 10.1002/hsr2.109 (PMC6375542; doi:10.1002/hsr2.109)
Supplement: Supplementary file 1 — Table S1. Time spent in activities requiring at least 2.5 METs [file HSR2-2-e109-s001.docx]

Supplementary table S1. Time spent in activities requiring at least 2.5 METs.

| Parameter | Patients with sarcoidosis  *N* = 53 | Healthy controls *N* = 28 | *P* value | Adjusted  *P* value^b^ |
| --- | --- | --- | --- | --- |
| Energy expenditure >2.5 METs, kcal/day [median (IQR)] | 481 (222–989) | 842 (598–1260) | < 0.001^a^ | 0.002^a^ |
| Duration of physical activity >2.5 METs, min/day [mean (SD)] | 152 (128) | 248 (118) | 0.001 | 0.023 |

^a^ After log transformation. ^b^ Calculated using multivariable linear regression analysis adjusted for age, sex, and body mass index.

Footnote. A cut-off of 2.5 METs was selected to facilitate comparison with the results of our study of patients with idiopathic pulmonary fibrosis (Wallaert et al., Physical activity in daily life of patients with fibrotic idiopathic interstitial pneumonia. [*Chest.*](https://www-ncbi-nlm-nih-gov.gate2.inist.fr/pubmed/23928896) 2013;144(5):1652-1658. doi: 10.1378/chest.13-0806; Wallaert et al., Effects of pulmonary rehabilitation on daily life physical activity of fibrotic idiopathic interstitial pneumonia patients. [*ERJ Open Res.*](https://www-ncbi-nlm-nih-gov.gate2.inist.fr/pubmed/29900176) 2018;4(2). pii: 00167-2017. doi: 10.1183/23120541.00167-2017. eCollection 2018 Apr).
